# Cobalt-Doped Carbon Nitride for Efficient Removal of *Microcystis aeruginosa* via Peroxymonosulfate Activation

**DOI:** 10.3390/toxins16110455

**Published:** 2024-10-24

**Authors:** Wen Yan, Chuqiao Li, Yunjuan Meng, Yao Yue, Teer Wen, Jiafeng Ding, Hangjun Zhang

**Affiliations:** 1 School of Life and Environmental Sciences, Hangzhou Normal University, Hangzhou 310018, China; 2022111010069@stu.hznu.edu.cn (W.Y.); 2023111010085@stu.hznu.edu.cn (C.L.); 20221010086@stu.hznu.edu.cn (Y.M.); 2023111010067@stu.hznu.edu.cn (Y.Y.); 2023111010099@stu.hznu.edu.cn (T.W.); 2 School of Engineering, Hangzhou Normal University, Hangzhou 310018, China; zhanghangjun@hznu.edu.cn; 3 Zhejiang Provincial Key Laboratory of Urban Wetlands and Regional Change, Hangzhou 311121, China

**Keywords:** *Microcystis aeruginosa*, persulfate, 2CoCN, singlet oxygen, oxidative stress, MC-LR

## Abstract

Heterogeneous persulfate activation is an advanced technology for treating harmful algae in drinking water sources, while it remains a significant hurdle in the efficient management of cyanobacterial blooms. In this study, super-dispersed cobalt-doped carbon nitride (2CoCN) was prepared to activate peroxymonosulfate (PMS) for simultaneous *Microcystis aeruginosa* inhibition and microcystin (MC-LR) degradation. When the initial PMS and 2CoCN concentrations were 0.3 g/L and 0.4 g/L, respectively, the efficiency of algal cell removal reached 97% in 15 min, and the degradation of MC-LR reached 96%. Analyses by SEM, TEM, and EEM spectra revealed that the reaction led to changes in algal cell morphology, damage to the cell membrane and cell wall, and the diffusion of thylakoid membranes and liposomes. The activities of antioxidant enzymes (superoxide dismutase and catalase) and antioxidants (glutathione) in algal cells generally increased, and the content of malondialdehyde increased, indicating severe damage to the cell membrane. Radical capture experiments confirmed that singlet oxygen (^1^O₂) was the key species destroying algal cells in the 2CoCN/PMS system. The 2CoCN/PMS system was effective in removing *M. aeruginosa* within a wide pH range (3–9), and 2CoCN had good reusability. Additionally, three degradation products of MC-LR were identified by LC–MS/MS analysis, and a possible mechanism for the inactivation of *M. aeruginosa* and the degradation of MC-LR was proposed. In conclusion, this study pioneered the 2CoCN/PMS system for inhibiting *M. aeruginosa* and degrading microcystin, aiming to advance water purification and algae removal technology.

## 1. Introduction

The worldwide prevalence of algal proliferations has been noted to severely disrupt aquatic ecological balance, contributing to the generation of harmful algal metabolites. These metabolites pose a substantial risk to human health, livestock, and aquatic ecosystems, thereby raising environmental concerns. *Microcystis aeruginosa* (*M. aeruginosa*) is the dominant species responsible for these blooms, which is known to produce microcystin compounds [1,2,3,4,5]. The prolonged consumption of water contaminated with microcystin can potentially induce hepatic and biliary tract abnormalities [6]. A large number of studies have confirmed that microbial degradation, chemical methods, and physical treatments are effective strategies to reduce cyanobacterial blooms [7]. Nonetheless, the practical implementation of these methods harbors certain shortcomings. For example, microbial degradation may be limited by environmental conditions, such as temperature and pH, resulting in unstable degradation efficiency [8]. Chemical methods may cause secondary pollution and potential harm to the environment [9]. Physical treatment methods may have high costs and limited effectiveness for large-scale cyanobacterial bloom treatment [10]. Thus, the development of methods is urgently warranted for simultaneously removing cyanobacteria and intracellular organic matter.

Recently, numerous novel technologies for disinfection and algae inactivation have arisen [11,12,13]. Advanced oxidation processes (AOPs) are a technology that oxidatively degrades pollutants by generating reactive oxygen species (ROS) such as hydroxyl radicals. Among the various AOPs, persulfate-based AOPs (PS-AOPs) have garnered substantial interest, owing to their exceptional oxidation capability (ranging from 2.5 to 3.1 V), prolonged lifetime (30–40 μs), and broad pH applicability (spanning 11 to 14). The effective activation of persulfate (PS) is achieved through a variety of heterogeneous catalysts, including metal oxides, metal-supported catalysts, metal-nonmetal hybrids, nano-carbon derivatives, and clay minerals [14,15,16]. Notably, changes in the valence of the metal catalyst enhance electron transfer, which in turn catalyzes the generation of substantial amounts of sulfate radicals (•SO_4_^−^) and hydroxyl radicals (•OH) from persulfate (PS) while producing only a small amount of singlet oxygen (^1^O_2_) [17]. The ^1^O_2_ species is stable at the sediment–water interface and is less affected by variations in water quality, reducing the chances of its quenching [18]. Currently, the investigation into the inactivation of algae via PS-AOP techniques predominantly centers on pre-oxidation flocculation methods utilizing UV activated persulfate (UV/PS) or Fe-activated persulfate (Fe/PS) systems [19]. Due to their reduced secondary pollution, heterogeneous activation methods garner greater interest compared to homogeneous activation. Consequently, further research is warranted into the inactivation of algae using PS-AOPs, particularly focusing on heterogeneous catalytic systems and their respective inactivation mechanisms.

Research has centered on the utilization of carbon materials impregnated with metal nanoparticles (NPs) in heterogeneous Fenton-type or analogous processes [20]. To enhance the metal utilization efficiency, a practical approach involves downscaling the metal particles and integrating them atomically onto supportive substrates [20]. For instance, cobalt (Co), a transition metal, exhibits a favorable effect in activating PMS [21]. Nonetheless, optimizing the Co/PMS system necessitates immobilizing cobalt (Co) transition metal atoms onto an apt support that provides ample anchoring sites and robust metal-carrier bonding, thus enhancing Co site dispersion and stability while preventing Co particle aggregation. Previous studies have shown that the employment of Co-incorporating metal–organic framework (MOF) material as a Co precursor effectively boosts the dispersion of Co sites within the catalysts [21]. Graphitic carbon nitride (g-C_3_N_4_), an esteemed polymeric non-metallic semiconductor, is conducive to the formation of Co-Nx coordination complexes, thereby serving as an ideal substrate for stabilizing positively charged cobalt (Co) transition metal species [21]. Furthermore, g-C_3_N_4_ exhibits excellent physicochemical stability, robust photocatalytic efficiency under visible light, adjustable electronic configuration, cost-effectiveness, and environmental friendliness [22]. Accordingly, g-C_3_N_4_-based catalysts emerge as optimal AOP materials in water treatment. Utilizing the inexhaustible resource of visible light, g-C_3_N_4_ can be efficiently activated to generate electrons, which then promote the transition from Co^3+^ to Co^2+^, thereby accelerating the generation of ^•^OH, •SO_4_^−^, and ^1^O_2_ radicals and enhancing the removal efficiency of algal cells.

This study focuses on the synthesis of a highly dispersible 2CoCN composite, a novel material designed to enhance the activation of PMS in heterogeneous systems. The primary objective is to evaluate its effectiveness in not only inactivating *M. aeruginosa* but also in degrading the algal toxins it produces, which pose significant risks to aquatic ecosystems and human health. A series of optimization experiments were conducted to examine how varying dosage levels and pH affect the catalytic performance of the 2CoCN composite, as these parameters significantly influence reaction kinetics and the PMS activation process. This research further investigates the morphological and biological changes in algal cells after treatment, highlighting the oxidative stress induced by active oxygen species. The composition of algal suspensions was analyzed before and after treatment to assess the structural integrity and viability of the algal cells. Advanced techniques were utilized to detect and quantify the active oxygen species generated and to examine free-radical quenching, essential for understanding the oxidative mechanisms involved. Additionally, an investigation into the mechanisms behind cyanobacterial inactivation and toxin degradation was conducted, focusing on the interactions between the 2CoCN composite and PMS, as well as the biochemical pathways that lead to the neutralization of harmful algal blooms. Overall, this study introduces an eco-friendly method to deactivate *M. aeruginosa* and degrade algal toxins, offering innovative strategies for managing harmful cyanobacterial blooms. The findings have potential applications in water treatment and environmental remediation.

## 2. Results and Discussion

### 2.1. Removal of M. aeruginosa and MC-LR by the 2CoCN/PMS System

**Figure 1:**
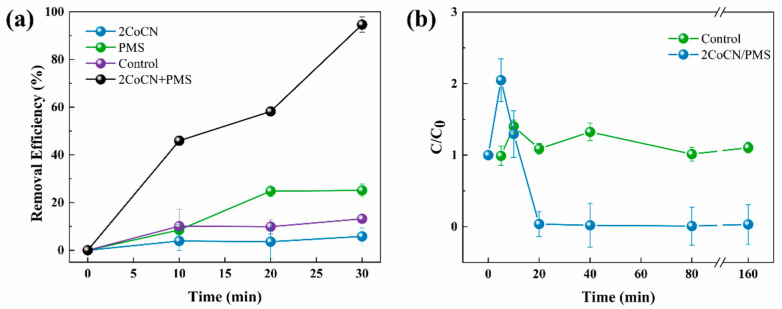
Variations of algal removal efficiency (**a**) and MC-LR concentrations (**b**) in the 2CoCN/PMS system. (C/C₀ represents the concentration of MC-LR during the reaction divided by its initial concentration.) Error bars represent the standard deviations (*n* = 3).

As shown in Figure 1a, compared to the control group with no additions, the treatment with either PMS or 2CoCN alone did not significantly change the concentration of *Microcystis aeruginosa* cells at pH 8.5, maintaining it close to the original suspension’s level. The removal efficiency is 25% and 5% in 30 min, respectively. However, after the addition of PMS (0.45 g/L) and 2CoCN (0.40 g/L), over the initial 10 min, the removal efficiency for M. aeruginosa cells progressively attained 45%. Subsequently, the solution rapidly attained transparency and clarity, with minimal suspended cells, achieving a removal efficiency of 94%, thereby demonstrating its capacity to efficiently eliminate *Microcystis aeruginosa* within 30 min. The results were mainly attributed to that the 2CoCN/PMS system may strongly release reactive oxygen species (ROS), which could significantly contribute to the deactivation of cyanobacterial cells. Similar to this study, Yu et al. [23,24] found that the single-iron-doped graphite/PMS system can efficiently product ROS to inactivate four harmful cyanobacteria.

The 2CoCN/PMS system also evaluated the degradation of algal toxins (MC-LR) to mitigate the harmful effects of algal toxins released due to cell rupture. The changes in the MC-LR concentration, as a function of reaction time, are shown in Figure 1b. At the first 5 min, the concentration of MC-LR surpassed its initial level due to the rupture of numerous algal cells. Subsequently, within the 2CoCN/PMS system, the MC-LR concentration underwent a gradual decline. At last, the liberated concentration of MC-LR was degraded to approximately 96% after a duration of 40 min. More importantly, the concentration of MC-LR did not increase over the subsequent 120 min; this suggests that the algal cells may have ruptured in large numbers at 30 min and that MC-LR was also being removed synchronously. It has also been demonstrated in our previous studies that MC-LR can effectively degrade into smaller molecules [25].

**Figure 2:**
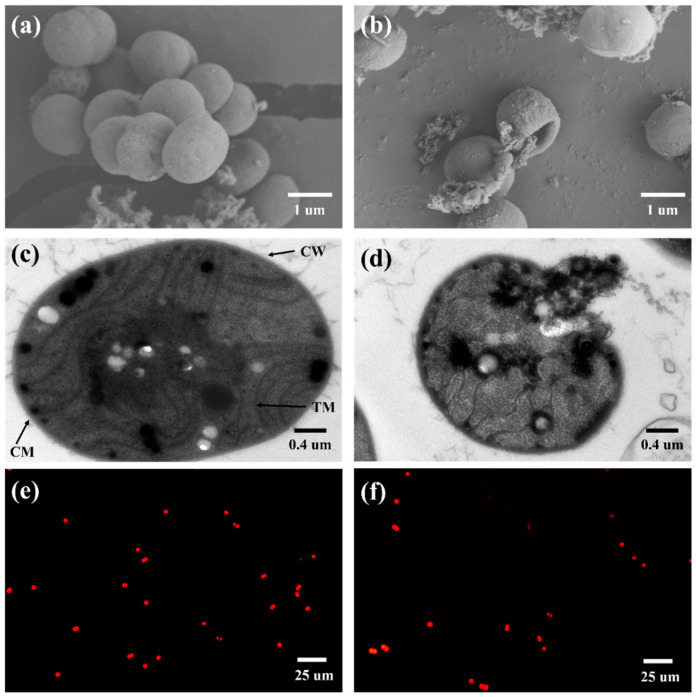
The SEM images of surface morphology of *M. aeruginosa*: before (**a**) and after reaction (**b**); the TEM images: before (**c**) and after reaction (**d**); chlorophyll a spontaneous fluorescence intensity: before (**e**) and after reaction (**f**).

To elucidate the impact of ROS generated in the 2CoCN/PMS system on cellular morphology, we employed SEM and TEM to examine the extracellular morphological alterations of *Microcystis aeruginosa* cells. In the control group (Figure 2a), the *Microcystis aeruginosa* cells exhibited a regular spherical shape and an intact smooth surface at 0 min, which were accompanied by natural metabolites such as extracellular polymeric substances (EPSs). Similarly, the algae cell exhibited a normal morphology, with intact cellular membranes (CMs) and cell walls (CWs) in Figure 2c. In this state, a regular arrangement of thylakoid membranes (TMs) was clearly visible, with a uniform distribution of cytoplasm and a normal central nucleoid region. As the reaction proceeds, the cell membrane folds inward. After 30 min of reaction, the cell membrane folds inward severely in Figure 2b. It suggests that ROS attacks the cell membrane, causing it to deform and fold. Moreover, as illustrated in Figure 2d, following the reaction, both the cellular membrane and cell wall sustained damage, resulting in the diffusion of thylakoid membranes and liposomes outside the cell. Such alterations may lead to the release of algal toxins, exerting adverse effects on the surrounding environment [26]. It was demonstrated in a similar study that ROS can penetrate into algal cells, triggering their release of algal toxins [25]. Additionally, the 2CoCN catalyst demonstrated proficiency in adhering to the surface of algal cells, enabling the generated reactive species to efficiently compromise the cell membranes, ultimately leading to the inactivation of the algal cells and the disintegration of extracellular and intracellular components. The spontaneous fluorescence intensity of chlorophyll a can serve as one of the important activity indicators of *M. aeruginosa*. Compared to 0 min in Figure 2e, a notable alteration in the fluorescence intensity of algal cells was observed after 30 min, indicating the demise of a substantial number of algal cells in Figure 2f. In our previous experiment, we observed a distinct cell outline with intense fluorescence, indicating that the captured cells remained intact by amorphous carbon-based zinc oxide [27].

**Figure 3:**
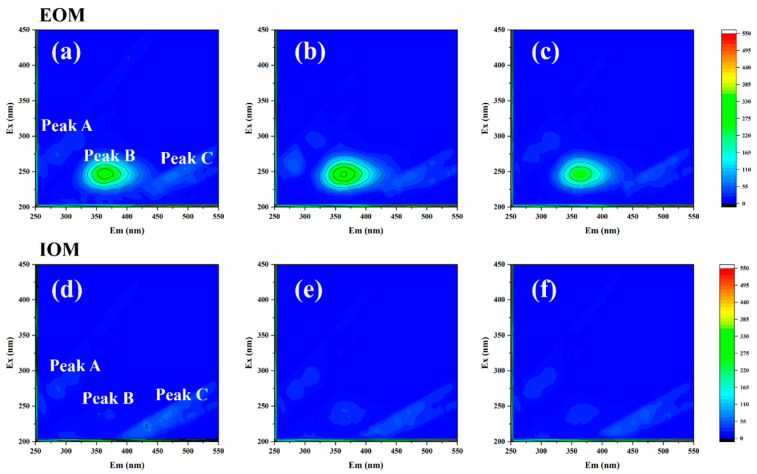
The excitation emission matrix spectrum of EOM and IOM; EOM: 0 min (**a**), 15 min (**b**), 30 min (**c**); IOM: 0 min (**d**), 15 min (**e**), 30 min (**f**).

Excitation–emission matrix (EEM) spectra were utilized to analyze the chemical composition and variations in algal organic matter (AOM) during the inactivation of algae by 2CoCN/PMS. As shown in Figure 3, three distinct fluorescence peaks have been identified, specifically at λex/λem = 275/320 nm (designated as peak A), 220/320 nm (peak B), and 275/450 nm (peak C). These peaks correspond to dissolved microbial metabolites, aromatic proteins (consisting of tyrosine and tryptophan), and fulvic-acid-like substances, respectively [28]. The potential source of humic-acid-like and fulvic-acid-like substances could be attributed to the oxidative decomposition of macromolecular organic matter or naturally occurring apoptotic algal cells [29]. The significant intensities of peaks A and B in the EOM spectrum indicate the rapid release of dissolved microbial metabolites and aromatic proteins within 15 min due to cellular damage. However, the intensity of these peaks decreased at 30 min, possibly because the 2CoCN/PMS system degraded EOM. Peak A of IOM increased at 15 min, probably due to some stress response of the algae that promoted the production of aromatic proteins [25]. Same as EOM, all peak intensities decreased at 30 min; this phenomenon can be ascribed to a reduction in the quantity of algal cells or to the direct action of 2CoCN/PMS on these substances. Based on the aforementioned findings, it is evident that the 2CoCN/PMS system effectively facilitates the catalytic inactivation of *M. aeruginosa*, along with the subsequent degradation of AOM.

### 2.2. Influence Factors for Algae Removal Efficiency

**Figure 4:**
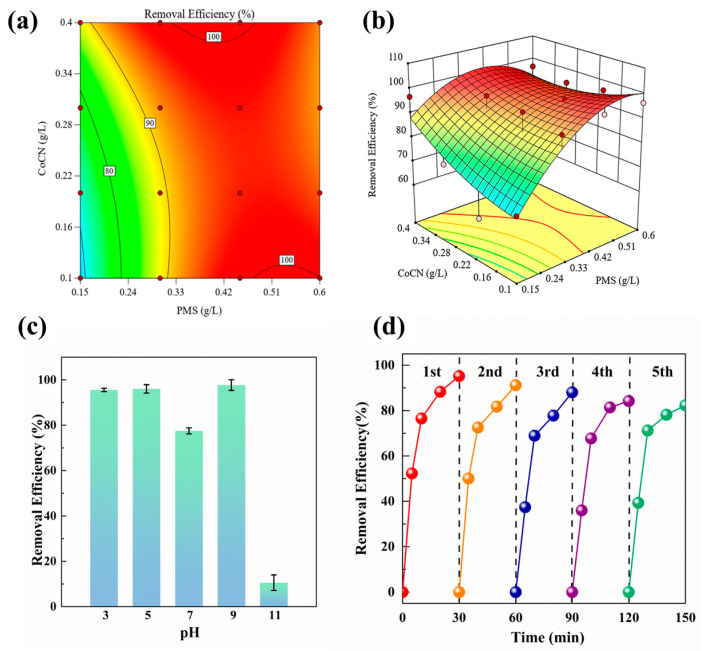
Response surface (**a**) and 3D contour plot (**b**) of interactive effect between the different factors for the removal of algal cells; typical time courses for algae removal in different pH (**c**); recyclability experiment (**d**).

A series of batch experiments were systematically conducted under varying pH conditions to investigate the impact of acidity and alkalinity on the removal efficiency of *M. aeruginosa*. The findings indicate that within neutral and mildly acidic environments, the 2CoCN/PMS system maintains a consistently high level of removal efficiency, as evident in Figure 4c. Notably, this pH range is broader compared to homogeneous activation methods, suggesting a greater adaptability of the 2CoCN/PMS system under diverse water conditions [30]. However, under alkaline conditions, specifically when the pH exceeds 11.0, a significant reduction in the removal effect was observed. This decrement is attributed to the decreased oxidation capability of PMS under alkaline conditions, as reported in previous studies [19]. In summary, the 2CoCN/PMS system demonstrates the effective removal of *M. aeruginosa* within a pH range of 3–9. This system is applicable for the inactivation of *M. aeruginosa* within a pH range spanning from 3.0 to 9.0. However, the actual aquatic environments where algae thrive are often alkaline [31], thus indicating the potential of the 2CoCN/PMS system for practical application in the control of algal blooms. 

Figure 4a,b demonstrated the synergistic impact of varying concentrations of 2CoCN and PMS on the removal of *M. aeruginosa*. As the dosage of 2CoCN (or PMS) increased from 0.1 g∙L^−1^ (0.15 g∙L^−1^) to 0.40 g L^−1^ (0.60 g∙L^−1^), the removal of *M. aeruginosa* increased from 6% to 98%. This underscores the reliance of algae removal efficiency on the combined effects of PMS and 2CoCN. At lower 2CoCN concentrations, PMS activation is presumably hindered, while at lower PMS concentrations, despite effective activation, fewer free radicals are generated for optimal algae removal. Furthermore, when PMS dosage was raised from 0.3 g∙L^−1^ to 0.6 g∙L^−1^ under identical conditions, the removal efficiency reached a saturation point, thus indicating an optimal dosage of 0.3 g∙L^−1^ for both 2CoCN and PMS. 

The potential applicability of 2CoCN is contingent upon its reusability, a crucial parameter that has been assessed accordingly. The experimental results indicate that the algae removal effect will not significantly decrease after five consecutive cycles of recycling and reuse (Figure 4d). This result indicates that 2CoCN maintains good reusability. 

### 2.3. Oxidative Stress Indicators and Proteins

**Figure 5:**
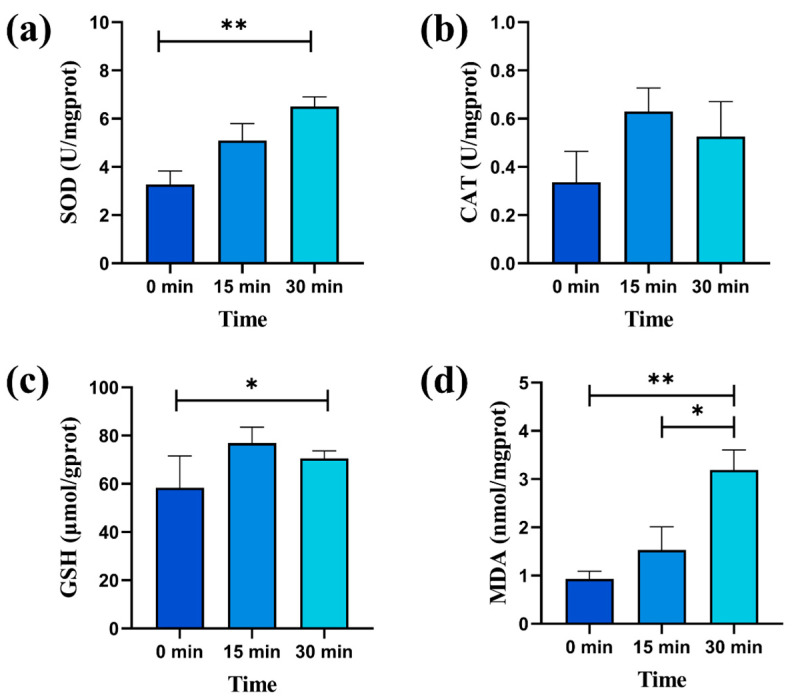
Oxidative stress indicators after 30 min of reaction: SOD (**a**), CAT (**b**), GSH (**c**), and MDA (**d**); (* *p* < 0.05, ** *p* < 0.01).

To investigate the antioxidant defense mechanisms demonstrated by *M. aeruginosa* in response to the 2CoCN/PMS system, we measured antioxidant defense enzymes (superoxide dismutase and catalase) and antioxidants (glutathione) [32]. When confronted with adverse environmental stress, microalgae trigger the production of substantial ROS within their cells, leading to severe oxidative damage. This damage can progress to cellular structural impairment, membrane rupture, and, ultimately, the leakage of cellular contents [33]. To counteract this excessive ROS generation, algal cells produce and accumulate antioxidant enzymes and antioxidants, thereby protecting themselves from oxidative damage [34]. Figure 5a–c correspond to the effects of the 2CoCN/PMS system on the superoxide dismutase (SOD), catalase (CAT) and glutathione (GSH) activities of *M. aeruginosa*, respectively. The activities of SOD, GSH, and CAT all exhibited an overall upward trend after the reaction. Specifically, GSH and CAT reached their peak activities at 15 min and then declined after 30 min. However, the activity of SOD peaked after 30 min of the reaction, reaching 1.98 times higher than that before the reaction. This indicates that under environmental stress, the antioxidant stress defense system of *M. aeruginosa*, including SOD, GSH, and CAT, undergoes certain destruction as a response to the increased stress [35].

Lipids are biomacromolecules that play a crucial role in cellular physiological activities, and changes in cellular lipid content can reflect the physiological state of algal cells [36]. Malondialdehyde (MDA) can reflect the level of lipid peroxidation, thereby indicating the level of oxidative damage to cell membranes [37]. In response to stress, the content of MDA continuously increased within 30 min, reaching 3.42 times higher than before the stress (Figure 5d), indicating severe damage to the cell membranes of *Microcystis aeruginosa* [38]. 

### 2.4. Mechanism and Functionality of ROS 

**Figure 6:**
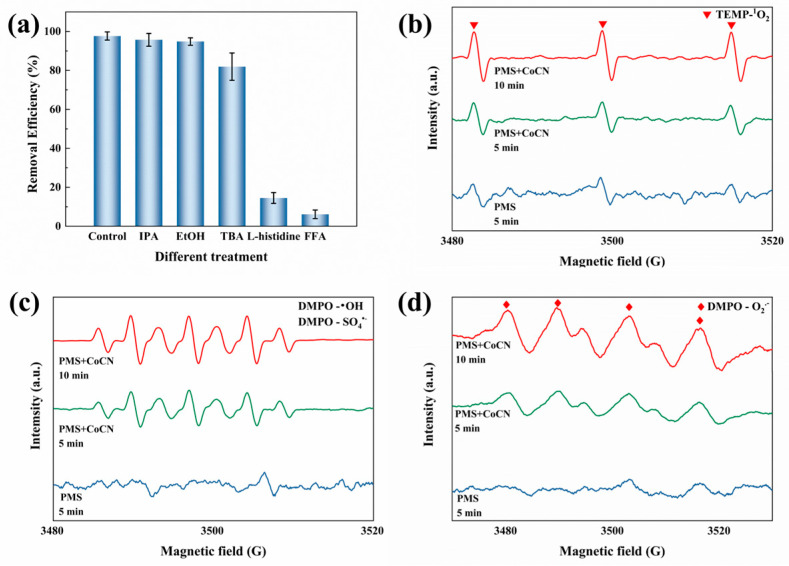
Radical scavenging experiment of algae removal in 2CoCN/PMS system (**a**); © reaction contains approximately 0.60 mg·L^−1^of chlorophyll *a*, 0.15 g·L^−1^of 2CoCN and 0.30 mM of PMS and 20 mM of the scavengers; EPR spectrum: ^1^O_2_ (**b**), O_2_^•−^ (**c**), SO_4_^•−^ and ^•^OH (**d**).

To gain a deeper understanding of the *M. aeruginosa* inactivation process, experiments focusing on ROS and hole trapping were devised to identify the key ROS involved in the degradation pathway. Consequently, to quench hydroxyl radicals (^•^OH), sulfate radicals (SO_4_^•−^), holes (*h*^+^), and singlet oxygen (^1^O_2_), specified concentrations (50 mM) of isopropanol (IPA), tert-butyl alcohol (TBA), ethanol (EtOH), furfuryl alcohol (FFA), and L-histidine were introduced [39,40,41]. Figure 6a demonstrates the quenching efficacy of various scavengers on free radicals. After quenching treatments with isopropanol, ethanol, tert-butanol, L-histidine, and furfuryl alcohol, the algal removal rates were 97%, 95%, 94%, 81%, 14%, and 6%, respectively. The removal of algal cells is evidently attributed to a significant contribution of ^1^O_2_. When L-histidine and FFA (as quenchers for ^1^O_2_) were added, the inactivation efficiency was significantly reduced. The reaction rate was also reduced when C_2_H_6_O and IPA were added. To further elucidate the production of reactive oxygen species (ROS), electron paramagnetic resonance (EPR) spectroscopy was employed for investigation during the activation of PMS by 2CoCN. TEMP is utilized as the scavenging agent for ^1^O_2_ (Figure 6b) and DMPO as the capture agent for O_2_^•−^, ^•^OH, and SO_4_^•−^ (Figure 6c,d). The 1:1:1 characteristic triple signal of TEMP-^1^O_2_ is clearly shown in Figure 6b. Meanwhile, the signals corresponding to DMPO—^•^OH and DMPO SO_4_^•−^—exhibit a characteristic ratio of 1:2:1:2:1:2:1, which is clearly shown in Figure 6c, this is due to the direct oxidation of DMPO by single-electron sources [42]. Additionally, the concentration of these free radicals progressively rises. The findings reinforce the notion that in the 2CoCN-activated PMS system, the inactivation of algal cells is primarily ascribed to ^1^O_2_.

**Figure 7:**
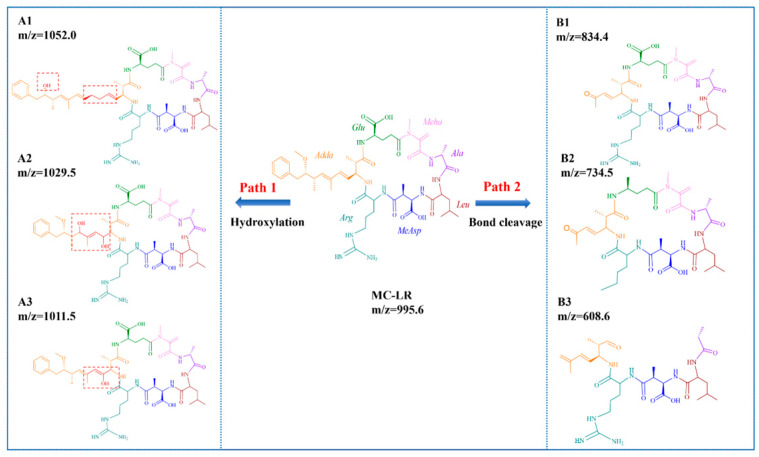
The possible degradation pathway of MC-LR in 2CoCN/PMS system.

Based on the comprehensive LC–MS/MS analysis, three distinct degradation products of MC-LR (*m/z* = 834.4, 734.5 and 608.6) were successfully identified and characterized. Figure 7 illustrates the potential degradation pathways of MC-LR within the 2CoCN/PMS system. MC-LR (*m/z* = 995.6), a biologically active cyclic heptapeptide, consists of the amino acid sequence comprising *Adda*, *Glu*, *Mdha*, *Ala*, *Leu*, *MeAsp*, and *Arg*. It has been documented that the double bond within the *Adda* side-chain undergoes hydroxylation, leading to the formation of a dihydroxy intermediate, denoted as A1–1 (*m/z* = 1052.0), A1–2 (*m/z* = 1029.5), and A1–3 (*m/z* = 1011.5) [43]. Owing to bond cleavage, the intermediates persist in yielding ketone derivatives B1-1 (*m/z* = 834.4) and B1-2 (*m/z* = 734.5) [44]. As the bond continues to break, the intermediates continue to produce ketone derivatives C1-1 (*m/z* = 608.6) [43].

**Figure 8:**
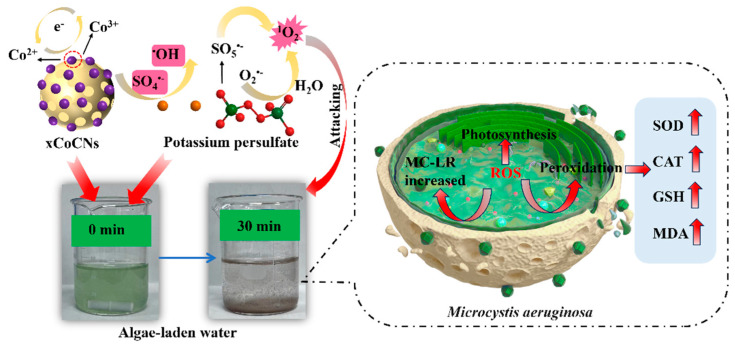
Possible mechanisms of homogeneous/heterogeneous ROS−flocculation process for *M. aeruginosa* removal in 2CoCN/PMS system.

A plausible mechanism for the inactivation of *M. aeruginosa* and MC-LR in the 2CoCN/PMS system is presented in Figure 8. The generation pathways of superoxide anions (O_2_^•−^), sulfate radicals (SO_4_^•−^), and singlet oxygen (^1^O_2_) are depicted in the figure as follows. In persulfate-mediated reactions, the disruption of –O–O– bonds can lead to the formation of free radicals such as hydroxyl (^•^OH) and sulfate (SO_4_^•−^) [41]. The continual transformation of two valence states of Co ions on the surface of the 2CoCN catalyst allows for the possible generation of ^1^O_2_ through two pathways, specifically, either through direct oxidation or recombination of O_2_^•−^, or through the oxidation of SO_5_^•−^.

## 3. Materials and Methods

### 3.1. Chemicals and Algal

All the chemicals used in this work were of HPLC grade. Urea (CH_4_N_2_O) and cobalt nitrate hexahydrate (Co(NO_3_)_2_·6H_2_O) were obtained from Aladdin Reagent (Shanghai, China). Potassium monopersulfate triple salt (K_5_H_3_S_4_O_18_ [42–46% KHSO_5_ basis]) and L-histidine (C_6_H_9_N_3_O_2_) were obtained from Macklin (China). Acetonitrile (CH_3_CN, gradient grade for liquid chromatography) and methanol (CH_4_O, ≤100%, gradient grade for liquid chromatography) were purchased from Merck (Darmstadt, Germany). H_2_SO_4_, NaOH, HNO_3_, pentanediol (C_5_H_12_O_2_), ethanol (C_2_H_6_O and EtOH), isopropanol (IPA), furfuryl alcohol, and tert-butanol were obtained from Sinopharm Chemical Reagent Co. Ltd. (Beijing, China). Ultrapure water with a resistance of 18.2 MΩ cm was prepared using a water purification device (Direct-Q 3UV, Darmstadt, Germany). 

*M. aeruginosa* (NO. FACHB-905) was provided by the Institute of Hydrobiology, Chinese Academy of Sciences (Wuhan, Hubei, China). The cells were cultured in BG11 medium and placed in an artificial climate incubator (HP1500GS, Shanghai Jingsheng Instrument Co., Ltd., Shanghai, China) at room temperature under a light intensity of 3000 lux and a light–dark ratio of 14 h:10 h. The algae cultures were shaken regularly every day.

### 3.2. Synthesis of 2CoCN Catalysts

Utilizing a one-step synthesis approach, super-dispersed cobalt-doped carbon nitride (abbreviated as 2CoCN) was successfully fabricated. Briefly, CoNO_3_·6H_2_O (2.0 mmol) and urea (10 g) were dissolved in ultrapure water and subsequently transferred into a 50 mL alumina crucible. Meanwhile, the mixtures were ultrasonicated individually for 1 h and subsequently dried at 70 °C for 24 h to yield the precursor. The alumina crucible was heated to 520 °C at a rate of 5 °C/min for 2 h. Once cooled to room temperature, the products were pulverized to powder form. This powder was washed three times with 0.1 M H_2_SO_4_, ethanol (EtOH), and ultrapure water, followed by drying at 60 °C for 48 h. 

### 3.3. Experimental Procedures

Another set of diluted algal suspension was prepared with an initial cell density of 3.2 × 10^6^ cells·mL^−1^. Ahead of experimentation, the pH of the algal solution was calibrated to 8.5 by 0.1 M of H_2_SO_4_ and 0.1 M of NaOH. The predetermined quantities of 2CoCN (0.015 g) and PMS (0.015 g) were incorporated into the prepared 50 mL algal solution and thoroughly agitated. Following the resuspension of the algal solution, sampling was conducted. Subsequently, a high-speed freezing centrifuge (KDC-140HR) was utilized to centrifuge the solution at 4 °C and 4000 rpm for 10 min, yielding the algal precipitate for further analysis. To derive the mean and its associated standard deviation, triplicate parallel trials were executed.

To establish the optimal concentrations of 2CoCN and PMS, orthogonal test groups were arranged, encompassing 2CoCN concentrations spanning 0.10 to 0.40 g L^−1^ and PMS concentrations ranging from 0.15 to 0.60 g L^−1^. To assess the influence of pH on inactivation efficiency, a set of experiments was undertaken at diverse initial pH levels, encompassing 3.0, 5.0, 6.0, 7.0, 9.0, and 11.0. The reusability of 2CoCN was evaluated through intermittent experiments (5 times). A 0.45 μm filter was used to capture and retain the catalysts for every reaction. Prior to reuse, the filtrate was washed multiple times in a mixture of toluene and n-hexane (*v*/*v* = 1:1) as well as distilled water. To quench SO_4_^•−^, ^•^OH, and ^1^O_2_ radicals, a concentration of 20.0 mM was used for the addition of TBA, MeOH, IPA, C_2_H_6_O, FFA, and L-histidine. Furthermore, to lyse algae cells resuspended in 0.5M phosphate buffered saline, we utilized an ultrasonic homogenizer (SCIENTZ-IID) at 500W intervals for a duration of 7 min. Then, we checked the oxidative stress and phycobiliprotein levels in the supernatant obtained after centrifugation. The concentrations of CAT (A007-1-1), SOD (A001-3), MDA (A003-1), GSH (A006-2-1), and total protein (TP; A045-2) were assessed employing techniques outlined by the Nanjing Jiancheng Biotechnology Research Institute located in China.

### 3.4. Analytical Methods

#### 3.4.1. Determination of Chla Removal Efficiency

To extract chlorophyll *a* (Chla) from the algal precipitate, a 95% ethanol solution was administered, followed by the determination of absorbance at 665 and 649 nm using a UV spectrophotometer (UV-1800, SHIMADZU). The Chla concentration was calculated using the following formula [45]:

Chla = 13.7 A_665_ − 5.76A_649_

The formula for calculating the removal efficiency of chlorophyll *a* is presented as follows:

R = (Chla_1_ − Chla_2_)/Chla_0_ × 100%

In this context, Chla1 represents the initial content of chlorophyll a, while Chla2 denotes its content at t minutes.

#### 3.4.2. Observation of Algae Cell Morphology

The *M. aeruginosa* suspension was centrifuged at 8000 rpm for 10 min before the supernatant was discarded. The collected algae samples were mixed with 2% glutaraldehyde for 4 h and then rinsed using a phosphate buffer solution for 15 min. The rinsing was repeated three times. Subsequently, the algae cells were dehydrated using different concentrations of ethanol (30%, 50%, 70%, 80%, 90%, and 100%) for 10 min each with gentle agitation. The dehydrated M. aeruginosa cells were dried by a vacuum freeze dryer and mounted on a copper stub to coat with gold for further SEM and TEM analysis.

#### 3.4.3. The Excitation Emission Matrix Spectra

Following centrifugation, the supernatant was passed through a 0.45 µm aqueous phase filter membrane to obtain the solution for subsequent measurement. The fluorescence spectrophotometer was utilized to analyze the prepared solution, resulting in the acquisition of the excitation–emission matrix (EEM) spectrum. The operational parameters were set as follows: excitation and emission slits of 10 nm, PMT voltage adjusted to 700 V, scanning speed set at 12,000 nm min^−1^, and the emission and excitation wavelength ranges spanning 200–550 nm [46].

#### 3.4.4. Analysis of MC-LR and Its Degradation Byproducts

The extraction cartridge (Oasis) was utilized to extract the samples. Post-conditioning, rinsing, and recovery steps, the instrument was dried with nitrogen gas (MTN-2800D, Tianjin Automation Scientific Instrument Co., Ltd., Tianjin, China) [47,48]. The solution was re-dissolved in methanol and finally filtered through a PTFE membrane (0.22 μm, Shanghai Aladdin Biochemical, Shanghai, China) for refinement. Subsequently, it was analyzed using UPLC–MS/MS instrumentation (Xevo TQ-S, Waters, Milford, MA, USA). The solvent system comprised a blend of water and acetonitrile at a volumetric ratio of 65:35. At the same time, intermediate degradation products of MC-LR, exhibiting *m/z* values spanning from 200 to 1000, were identified through continuous MS scanning mode within a range of 0 to 20 min.

## 4. Conclusions

The objective of this study was to establish the 2CoCN/PMS system for the mitigation of detrimental algal bloom effects in drinking water resources, emphasizing the concurrent deactivation of *M. aeruginosa* and the removal of MC-LR. In this study, super-dispersed 2CoCN was prepared to activate PMS for simultaneous *M. aeruginosa* inhibition and MC-LR degradation. Under optimal conditions, the inhibition efficiency of algal cells reached 97%. Despite reaction-induced oxidative stress damaging cell membranes, a 96% degradation rate for MC-LR was observed after 40 min. Both algal cell inhibition and MC-LR degradation were achieved. Furthermore, the physiological reaction of algal cells to environmental stress was thoroughly investigated, and a proposed mechanism for free-radical generation and MC-LR degradation was outlined. Through rigorous free-radical capture experiments, it was conclusively verified that singlet oxygen (^1^O₂) was the vital species destroying algal cells in the 2CoCN/PMS system. Overall, this study pioneers the 2CoCN/PMS system for *M. aeruginosa* inhibition and microcystin degradation, aiming to advance water purification and algae removal technology.

## Data Availability

All data supporting the results can be found within the manuscript.
